# Supplementary figures and images for: Modeling epistasis in mice and yeast using the proportion of two or more distinct genetic backgrounds: Evidence for “polygenic epistasis”
Source: PLoS Genet. 2020 Oct 26;16(10):e1009165. doi: 10.1371/journal.pgen.1009165 (PMC7644088; doi:10.1371/journal.pgen.1009165)

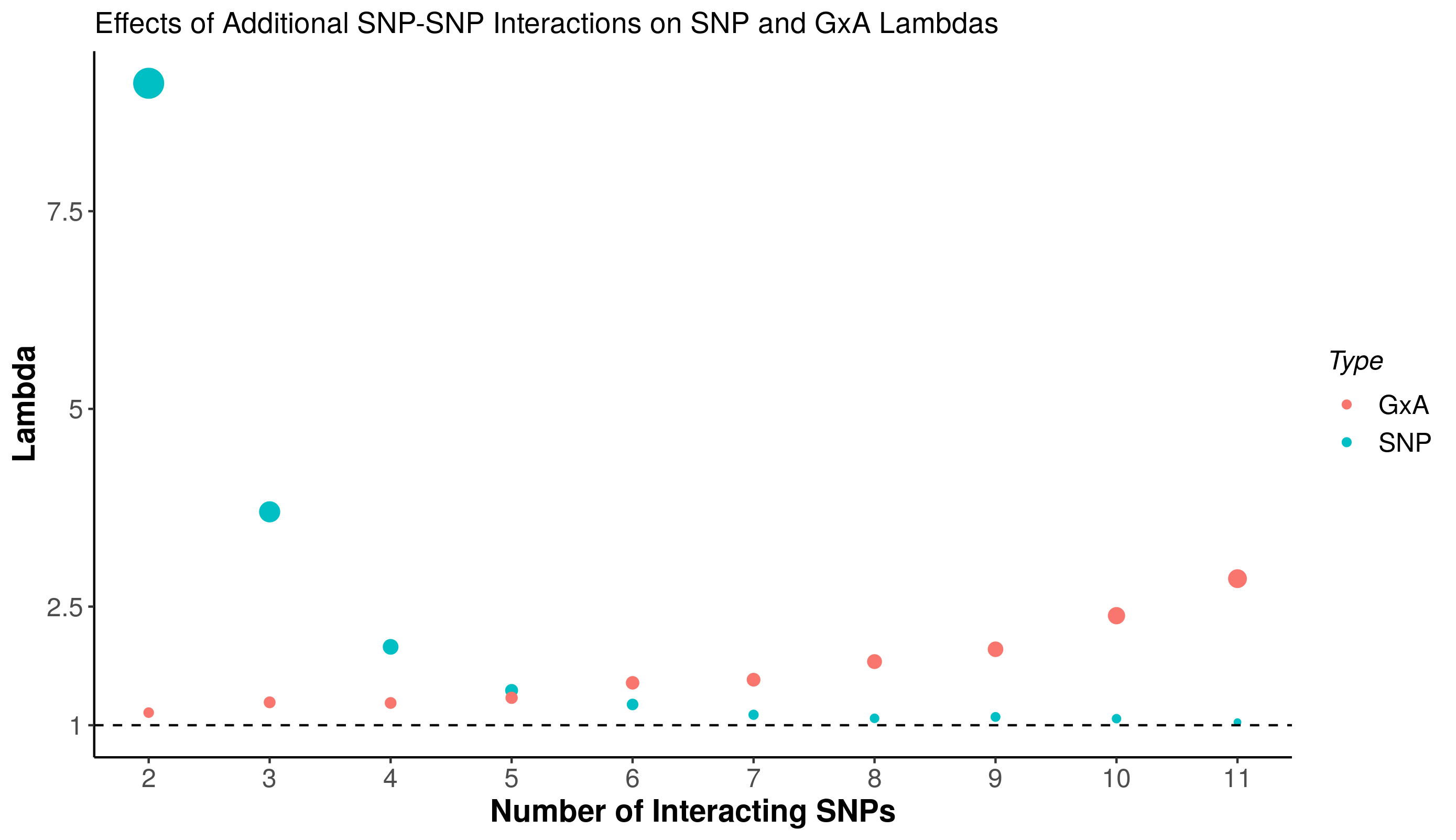

Supplement: S1 Fig — (TIF) [file pgen.1009165.s001.tif]
